# *CDKN2A* Deletion Leading to Hematogenous Metastasis of Human Gastric Carcinoma

**DOI:** 10.3389/fonc.2021.801219

**Published:** 2021-12-23

**Authors:** Juanli Qiao, Yuan Tian, Xiaojing Cheng, Zhaojun Liu, Jing Zhou, Liankun Gu, Baozhen Zhang, Lianhai Zhang, Jiafu Ji, Rui Xing, Dajun Deng

**Affiliations:** ^1^ Key Laboratory of Carcinogenesis and Translational Research (Ministry of Education/Beijing), Division of Etiology, Peking University Cancer Hospital and Institute, Beijing, China; ^2^ Key Laboratory of Carcinogenesis and Translational Research (Ministry of Education/Beijing), Department of Gastrointestinal Surgery, Peking University Cancer Hospital and Institute, Beijing, China; ^3^ Key Laboratory of Carcinogenesis and Translational Research (Ministry of Education/Beijing), Department of Tumor Biology, Peking University Cancer Hospital and Institute, Beijing, China

**Keywords:** *CDKN2A*, somatic copy number deletion, gastric carcinoma, metastasis, apoptosis

## Abstract

**Introduction:**

Somatic copy number deletion (SCND) of *CDKN2A* gene is the most frequent event in cancer genomes. Whether *CDKN2A* SCND drives human cancer metastasis is far from clear. Hematogenous metastasis is the main reason of human gastric carcinoma (GC) death. Thus, prediction GC metastasis is eagerly awaited.

**Method:**

GC patients (*n*=408) enrolled in both a cross-sectional and a prospective cohorts were analysed. *CDKN2A* SCND was detected with a quantitative PCR assay (P16-Light). Association of *CDKN2A* SCND and GC metastasis was evaluated. Effect of *CDKN2A* SCND by CRISPR/Cas9 on biological behaviors of cancer cells was also studied.

**Results:**

*CDKN2A* SCND was detected in 38.9% of GCs from patients (*n*=234) enrolled in the cross-sectional cohort. Association analysis showed that more *CDKN2A* SCND was recognized in GCs with hematogenous metastasis than those without (66.7% *vs.* 35.7%, *p*=0.014). *CDKN2A* SCND was detected in 36.8% of baseline pN_0_M_0_ GCs from patients (*n*=174) enrolled in the prospective study, the relationship between *CDKN2A* SCND and hematogenous metastasis throughout the follow-up period (62.7 months in *median*) was also significant (66.7% *vs.* 34.6%, *p*=0.016). Using *CDKN2A* SCND as a biomarker for predicting hematogenous metastasis of GCs, the prediction sensitivity and specificity were 66.7% and 65.4%. The results of functional experiments indicated that *CDKN2A* SCND could obviously downregulate P53 expression that consequently inhibited the apoptosis of MGC803 GC and HEK293T cells. This may account for hematogenous metastasis of GCs by *CDKN2A* SCND.

**Conclusion:**

*CDKN2A* SCND may drive GC metastasis and could be used as a predictor for hematogenous metastasis of GCs.

## Introduction

Gastric carcinoma (GC) is the third leading cause of cancer-related death worldwide (1). Distant or hematogenous metastasis, lymphatic or peritoneal spreading, and local recurrence are the key reasons for the failure of surgical treatment for patients with resectable GCs (2). Among these, hematogenous metastasis to liver, lung, bone, or brain is responsible for the greatest mortality in GC patients. Although many efforts have been made to discover prognosis biomarkers for GC (3–13), a feasible biomarker for prediction of hematogenous metastasis of GC is still eagerly awaited.

Different transcription start sites are used to synthesize the human *P16^INK4a^* as well as *P14^ARF^* mRNAs from the *CDKN2A* gene on chromosome 9p21 (14); they share the same exon-2 and have different translation reading frames. In addition to their functions in apoptosis, cell cycle arrest and senescence, the P16^INK4a^ and P14^ARF^ proteins play important function in prophylaxis of cell replicative stress through the P16^INK4a^-CDK4/6-RB1 and P14^ARF^-MDM2-P53/P21^CIP1^-CDK2-RB1 pathways, respectively (15–18). The mutation of the *CDKN2A* gene in the germline can result in a significant risk of developing melanoma or pancreatic cancer (19–21). Recently, it was reported that inactivation of *Cdkn2a/p16^ink4a^* gene by CRISPR/Cas9 significantly favored lung metastasis of mouse non-small cell lung carcinoma transplanted subcutaneously and artificial inactivation of *CDKN2A* gene initiates the invasion of human melanoma cells *via BRN2* activation (22, 23). Several human malignancies are characterized by somatic copy number deletion (SCND) of the *CDKN2A* gene (24). However, whether the inactivation of the *CDKN2A* gene by SCND affects hematogenous metastasis of human cancers has not been reported previously.

Recently, we identified a 5.1-kb common deletion region (CDR) within the *CDKN2A/P16^INK4a^* gene from intron-2 to promoter in 92% of *CDKN2A*-deleted human malignancies. Current FISH approach to detect SCNVs is composed of a set of probes covering at least 50-kb (at least 30-kb) DNA sequence that is not suitable for detecting the copy number of the 5.1-kb *CDKN2A* CDR. Therefore, we have developed a CDR-specific assay termed P16-Light to quantitatively detect somatic copy number variations (SCNVs) of the *CDKN2A* gene, and validated the assay with whole genome sequencing data (25). In present study, we further studied association of *CDKN2A* SCNVs with hematogenous metastasis of GC in patients enrolled in a cross-sectional cohort and confirmed the association in patients enrolled in a prospective cohorts. A set of biological experiments were also carried out to establish the causal relationship between them.

## Materials and Methods

### Study Design

**Figure 1:**
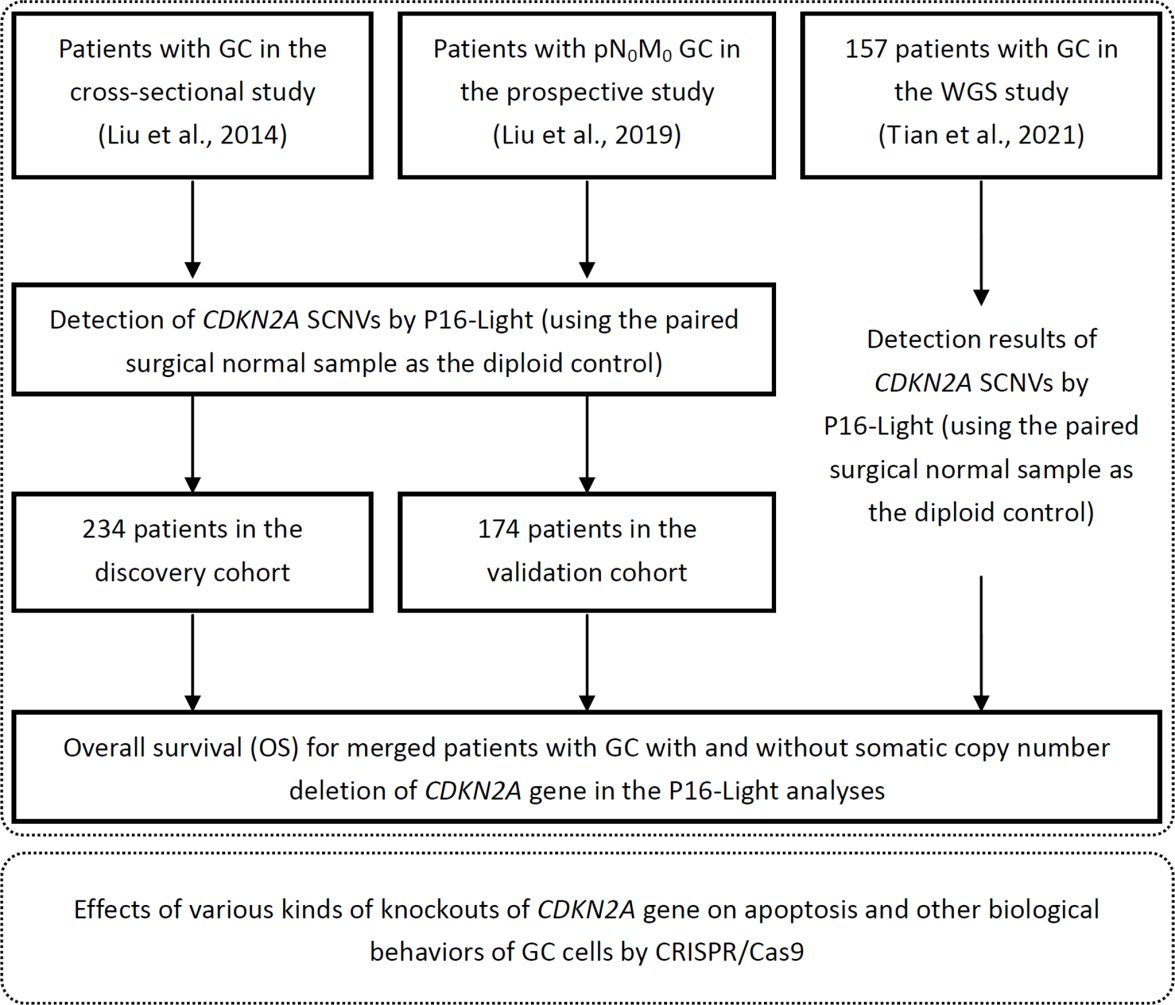
Working flow diagram. Clinical and biological studies were illustrated within top and bottom dashed line frames, respectively.

234 patients (from 1999 to 2003) enrolled in the cross-sectional study (26), and the other 174 patients (from 2002-2012) enrolled in a double-blind prospective study (NCT02159339) (27) with enough amounts of DNA samples for *CDKN2A* copy number analysis were included in the present study. Clinicopathological and follow-up metastasis/relapse information were collected from Peking University Cancer Hospital & Institute. Information on overall survival (OS) and *CDKN2A* SCND for 157 patients, who enrolled in our previous study (25, 28), were also included in the OS analysis as illustrated in **Figure 1**. The characterization of these GCs was done using the UICC-tumor-node-metastasis (TNM) approach from 2010 (29). Detailed information for each de-identified patient was listed in **Data File 1**.

### Preparation of DNA

Patients provided frozen fresh GC as well as paired surgical margin (SM) samples, which were collected and analysed. The phenol/chloroform technique was used to isolate the genomic DNA from these samples.

### Detection of *CDKN2A* SCNVs by P16-Light

P16-Light, a multiplex quantitative PCR assay using *GAPDH* as a reference gene, was performed according to our recent report (25). For the purposes of this study, each multiplex PCR was performed in a total volume of 20 μL, which consisted of an intron-2 probe of *CDKN2A* using forward and reverse primers of 10 μM each, probe for *GAPDH* using forward and reverse primers of 10 μM concentration, input DNA of 5-10 ng, and 10 μL of 2x TaqMan Universal Master Mix II of uracil-N-glycosylase (Kit-4440038, ABI, Lithuania) (**Table 1**). With the use of an ABI 7500 Fast Real-Time PCR System, three replicates of the PCRs were carried out in a MicroAmp Fast Optical 96-Well Reaction Plate with barcode (0.1 mL; ABI, China). For this particular PCR, the following criteria were used: an initial incubation for 10 min at 95°C, followed by 40 cycles at 95°C for 20 sec followed by 58°C for 60 sec. Using the *GAPDH* gene as a reference, the *CDKN2A* gene’s ΔCt value as well as relative copy number were computed. *CDKN2A* gene copy number deletion or amplification positive was identified when the average relative *CDKN2A* gene copy number in GC samples was substantially lower or greater than in the paired SM samples, respectively, in student t-test. As positive and negative controls, genomic DNA from A549 cells that did not include any *CDKN2A* alleles and genomic DNA from RKO cells that did have two wild-type *CDKN2A* alleles were used, respectively.

**Table 1:** Oligo sequences.

Gene	Assay	Oligo	Sequence	PCR product size
*CDKN2A*	P16-Light	F-primer	5’-caggtctgtttcctcatttg-3’	129-bp
		R-primer	5’-ggtcagattagttgagttgtg-3’	
		Probe	FAM-ctggctggaccaacctcagg-BHQ1	
*GAPDH*	P16-Light	F-primer	5’-gctcacatattctggaggag-3’	135-bp
		R-primer	5’-ggtcattgatggcaacaata-3’	
		Probe	Cy5-tgccttcttgcctcttgtctctt-BHQ2	
*CDKN2A*	CRISPR/Cas9	E1a_sgRNA	5’-ACCGTAACTATTCGGTGCGTtgg-3’	
		E1b_sgRNA	5’-GCACGCGCGCCGAATCCGGAggg-3’	
		CDR-gRNA#1	3’-CGTCAAAGTCGTCTGTCgac-5’	
		CDR-gRNA#2	3’-gtgGCTCTTAGCTTTAGTGG-5’	
		E2_sgRNA	5’-TCCCGGGCAGCGTCGTGCACggg-3’	
CDKN2A exon-1a	CRISPR/Cas9	E1a_oF	5’-cggtccctccagaggatttg-3’	411-bp
		E1a_oR	5’-ggagaatcgaagcgctacctg-3’	
CDKN2A exon-1b	CRISPR/Cas9	E1b_oF	5’-agtctgcagttaagggggcag-3’	312-bp
		E1b_oR	5’-gacttttcgagggcctttccta-3’	
CDKN2A exon-2	CRISPR/Cas9	E2_oF	5’-tgagggggctctacacaagc-3’	363-bp
		E2_oR	5’-tatgcgggcatggttactgc-3’	

### Cell Lines and Cultures

We used the RPMI-1640 medium to cultivate human gastric cancer cell line MGC803 and immortalized embryo kidney cell line HEK293T (cordially given by Dr. Yang Ke at Peking University Cancer Hospital and Professor Yasuhito Yuasa of Tokyo Medical and Dental University, respectively). Fetal bovine serum (FBS) was added to the medium at 10% (v/v). Beijing JianLian Genes Technology Co., Ltd. examined and certified these cell lines before they were utilized in this investigation. In this examination, analyse the Goldeneye™ 20A STR Identifiler PCR Amplification kit was used to evaluate STR patterns.

### Assays of Cell Proliferation, Migration, and Invasion With IncuCyte

In 96-well plates, cells were seeded with 2,000 cells per well and grown for a minimum of 96 hrs, with 10 wells per group. A long-term dynamic observation platform was employed to take pictures of the cells every 6 hrs and collected the necessary data (IncuCyte, Essen, MI, USA). It was determined how many cells were confluent analyse using the IncuCyte ZOOM programme (Essen, Ann Arbor, MI, USA). The cells were seeded into 96-well plates at a density of 25,000 cells per well and then cultivated for 24 hrs to allow for real-time movement and invasion tracking, as previously described. After establishing a wound mark, the cells were rinsed three times with PBS to remove any remaining debris. For the invasion test, 50 µL Matrigel (acquired from BD Bioscience, San Jose, CA) diluted with RPMI 1640 Medium at a ratio of 1:8 was added after the cells had been rinsed with PBS and grown for 30 min at 37°C before being removed. For at least 96 hrs, the cells were cultivated on a regular basis and imaged every 6 hrs. Calculation of relative wound width was done with the same programme.

### Disruption of *CDKN2A* Exon 1a, 1b, 2 or CDR With CRISPR/Cas9 Technology

Exon 1a, 1b and 2 of the *CDKN2A* gene were knocked out by single-guide RNA (sgRNA) approaches, while the CRISPR/Cas9 system was utilized to knock out the *CDKN2A* gene’s CDR region *via* a dual gRNA strategy (30). The sgRNAs were created over an online platform available at the website (http://crispr.mit.edu) and synthesized by Thermo Scientific, Inc., Rockford, IL, USA (**Table 1**). To express Cas9 in the Lenti-CRISPR-V2 vector, the sgRNAs were cloned into the *BsmBI* restriction site of lenti-CRISPR-V2 vector (Plasmid #52961, Addgene, Inc.). Next, HEK293FT cells were transfected with lentivirus encoding gRNA and Cas9, and the results were confirmed in the lab. It was 72 hrs after transfection that the viral supernatants were collected, and the viruses were employed to infect MGC803 or HEK293T cells with the 0.45 μm PVDF filter (Millipore, USA). For three days after the virus infection, the infected cells were submitted to puromycin selection for one week, and genomic DNA from the surviving cells was extracted and put to PCR amplification and sequencing using the primers (**Table 1**). The cells were then planted into 96-well plates in order to select for monoclonal cells, which were then purified. For the wild-type (WT) control, we used cells that had transfected with control vector that was devoid of Lenti-CRISPR-V2.

### *P16^INK4A^* Overexpression

*P16^INK4A^* overexpression pIRES2-EGFP vector was constructed as previously described (3) and used to transiently transfect MGC803 cells using XtremeGENE HP DNA Transfection Reagent (Roche, Mannheim, Germany).

### Western Blot

In order to obtain a protein lysate, cells were collected and lysed. Proteins were separated on a PVDF membrane using a 10% SDS-PAGE gel, which was then were transferred. With primary antibodies including anti-P16 (1:3000, Abcam, UK), anti-RB1 (1:2500, Abcam, UK), anti-Phospho-RB1 (Ser807/811) (1:2500, Cell Signaling Technology, UAS), anti-GAPDH (1:10000, Protein Tech, China), the membrane was incubated for 1 hr at room temperature after being blocked with 5% fat-free milk for a night at 4°C. This was followed by three rounds of PBST washing (PBS with 0.1% Tween 20). Incubation with the relevant horseradish peroxidase-conjugated goat anti-goat at 1:3000 for anti-P16, anti-RB1, and anti-Phospho-RB1 or anti-mouse IgG at 1:10000 for anti-GAPDH was performed at room temperature for 1 hr after rinsing the membrane with distilled water. Through the use of an Immobilon Western Chemiluminescent HRP Substrate kit, the signals were seen (WBKLS0500, Millipore, Billerica, USA).

### Cell Apoptosis and Death Analyses

Cells were seeded in six-well plates (2 × 10^5^ cells per well). A trypsin treatment was performed on the cells after 48 hrs, followed two washes with cold PBS. In accordance with the manufacturer’s instructions, they were tagged with annexin V-FITC and propidium iodide (PI) (Dojindo, Japan). A BD Accuri C6 flow cytometer was then used to evaluate the cells (BD Biosciences, USA). With the BD Accuri C6 software, the percentages of cells in early apoptosis (annexin V-positive, PI-negative) and late apoptosis/necrosis (annexin V- and PI-positive) were calculated.

### TCGA Patient Cohorts

Copy-number alterations of the *CDKN2A* gene in tissues from 10488 and 11226 cancer patients, clinical information, and survival datasets in the TCGA and MSKCC PanCancer projects were downloaded from cBioport (**Data Files 2**, **3**), respectively (6, 7, 31–34).

### Statistical Analysis

It was determined whether there was a relationship between somatic CDKN2A SCND and clinicopathological characteristics using chi-square testing. Log-rank tests were used to compare OS between groups. Kaplan-Meier analysis was utilized to calculate the OS of patient. Student t-test was utilized to the difference of relative copy number of *CDKN2A* between GC and SM samples. The prediction sensitivity was equal to ratio of number of *CDKN2A* SCND-positive GC patients with follow-up hematogenous metastasis to number of all of GC patients with follow-up hematogenous metastasis. The prediction specificity was equal to ratio of number of *CDKN2A* SCND-negative GC patients without follow-up hematogenous metastasis to number of all of GC patients without follow-up hematogenous metastasis. A *p*-value of less than <0.05 was considered statistically significant important in all of these tests.

## Results

### Basic Information of Patients

The basic information for 234 patients with GC in the cross-sectional study and 174 patients with pN_0_M_0_ GC in the prospective study were listed in **Table 2**. Twenty-four patients (median follow-up of 62.7 months) were found to have distant metastasis, including hematogenous metastasis in 12 patients (six to liver, two to lung, one to bone, one to brain, one to transsepmuscle, and one to abdomen skin) and lymphatic/peritoneal metastasis in 12 other patients (cohort 2, **Data File 1**).

**Table 2:** Association of somatic copy number variations (SCNVs) of the *CDKN2A* gene by P16-Light with clinicopathological characteristics of Chinese patients with gastric carcinoma (GC) included in a cross-sectional study and a prospective study.

		SCNVs of *CDKN2A* in GC patients (*n*=234) in the cross-sectional study (26)					SCNVs of *CDKN2A* in pN_0_M_0_ GC patients (*n*=174) in the prospective study (27)				
		Amp. (%)	Diploid (%)	Del. (%)	Chi- square	*p*-value	Amp. (%)	Diploid (%)	Del. (%)	Chi- square	*p*-value
Sex	Male	18 (10.8)	78 (47.0)	70 (42.2)	**2.997**	0.003*	20 (16.3)	54 (43.9)	49 (39.8)	2.634	0.105
	Female	11 (16.2)	36 (52.9)	21 (30.9)			13 (25.5)	23 (45.1)	15 (29.4)		
Age	≥60 yrs	15 (12.0)	58 (46.4)	52 (41.6)	0.528	0.446	15 (16.5)	43 (47.3)	33 (36.3)	0.129	0.709
	<60 yrs	14 (12.8)	56 (51.4)	39 (35.8)			18 (21.7)	34 (41.0)	31 (37.3)		
Location	Cardia	1 (3.1)	17 (53.1)	14 (43.8)	1.665	0.197	11 (18.6)	23 (39.0)	25 (42.4)	0.59	0.442
	Noncardia	28 (13.9)	97 (48.0)	77 (38.1)			22 (19.1)	54 (47.0)	39 (33.9)		
Differentiation	Mod./Well	8 (12.1)	26 (39.4)	32 (48.5)	2.014	0.156	12 (19.0)	21 (33.3)	30 (47.6)	1.737	0.188
	Poor	21 (12.5)	88 (52.4)	59 (35.1)			19 (18.1)	53 (50.5)	33 (31.4)		
pTNM stage	I	1 (4.8)	14 (66.7)	6 (28.6)	0.584	0.445	12 (24.0)	24 (48.0)	14 (28.0)	1.983	0.159
	II	5 (11.9)	25 (59.5)	12 (28.6)			19 (18.6)	43 (42.2)	40 (39.2)		
	III	10 (13.2)	35 (46.1)	31 (40.8)			2 (9.1)	13 (59.1)	7 (31.8)		
	IV	13 (13.7)	40 (42.1)	42 (44.2)							
Invasion	T_1-2_	2 (4.4)	26 (57.8)	17 (37.8)	0.643	0.423	12 (24.0)	24 (48.0)	14 (28.0)	3.140	0.076
	T_3-4_	27 (14.4)	88 (46.8)	73 (38.8)			21 (16.9)	50 (40.3)	53 (42.7)		
Baseline lymph	N_0_	7 (10.8)	34 (52.3)	24 (36.9)	0	0.997	33 (19.0)	77 (44.3)	64 (36.8)		
metastasis	N_1-x_	22 (13.1)	80 (47.6)	66 (39.3)							
Baseline distant	M_0_	24 (12.5)	103 (53.6)	65 (33.9)	**6.362**	**0.012**	33 (19.0)	77 (44.3)	64 (36.8)		
metastasis	M_1_	5 (11.9)	11 (26.2)	26 (61.9)							
Baseline hematogenous	Negative	26 (12.6)	107 (51.2)	74 (35.7)	**6.028**	**0.014**	33 (19.0)	77 (44.3)	64 (36.8)		
Metastasis	Positive	2 (8.3)	6 (25.0)	16 (66.7)							
Follow-up hematogenous	Negative						33 (20.3)	73 (45.1)	56 (34.6)	**5.817**	**0.016**
Metastasis	Positive						0	4 (33.3)	8 (66.7)		
(Total)		29 (12.4)	114 (48.7)	91 (38.9)			33 (19.0)	77 (44.3)	64 (36.8)		

*Bold values: statistically significant.

### *CDKN2A* SCND Increases Risk of Hematogenous Metastasis of GCs in the Cross-Sectional Cohort

To clarify whether *CDKN2A* SCND could drive GC metastasis, we analysed the prevalence of *CDKN2A* SCNVs by P16-Light among 234 GC patients enrolled in the cross-sectional study (26). *CDKN2A* SCND and amplification were found in 91 (38.9%) and 29 (12.4%) of the GCs tested, respectively (**Data File 1**, cohort 1). The incidence of *CDKN2A* SCND was significantly greater in GCs with distant or hematogenous metastasis than GCs without distant or hematogenous metastasis (Chi-square test, *p*=0.012 or 0.014; **Table 2**). More *CDKN2A* SCNDs were also detected in GCs of males than those of females (*p*=0.003).

### *CDKN2A* SCND Increases Risk of Hematogenous Metastasis of pN_0_M_0_ GCs in the Prospective Cohort

Then, the feasibility of using *CDKN2A* SCND as a biomarker for predicting hematogenous metastasis of GCs was further validated among 174 patients with baseline pN_0_M_0_ GC enrolled in the independent prospective study cohort (27). Once again, association analyses showed that *CDKN2A* SCND significantly increased the risk of hematogenous metastasis of GCs during the follow-up: *CDKN2A* SCND was found in 8 (66.7%) of these 12 GCs from patients with hematogenous metastasis and no *CDKN2A* amplification was found. However, for 162 GCs without hematogenous metastasis, *CDKN2A* SCND and amplification were respectively detected in 56 (34.6%) and 33 (20.3%) GCs (*p*=0.016; **Table 2**). Using *CDKN2A* SCND as a biomarker for predicting hematogenous metastasis of GCs, the prediction sensitivity and specificity were 66.7% (8/12) and 65.4% (106/162), respectively.

### Mining Public SCNV Datasets: *CDKN2A* SCND Increases the Risk of Distant Metastasis of Various Cancers

To explore whether *CDKN2A* SCND may also affect distant metastasis of other cancers, we further mined The Cancer Genome Atlas (TCGA) PanCancer SCNV datasets (**Data File 2**) (6, 7, 31, 32, 41). We found that the frequency of *CDKN2A* deletion was significantly and consistently associated with an increased risk of local invasion (*p*<0.001) and distant metastasis of various cancers without lymph metastasis (*p*<0.025; **Figure S1A**), especially for head and neck squamous cell carcinoma (HNSC), kidney clear cell carcinoma (KIRC), pancreas adenocarcinoma (PAAD), skin cutaneous melanoma (SKCM), and stomach adenocarcinoma (STAD/GC) (**Figure S1B**). Mesothelioma (MESO) is an exception: significantly more *CDKN2A* deletion was detected in non-metastatic MESO than net distant metastatic MESO (*p*=0.045). Consistency with our above results, such relationships could not be observed among patients with lymph metastatic cancers (**Figures S1C, D**).

### *CDKN2A* SCND Correlates With Short OS of Patients With GC and Other Cancers

**Figure 2:**
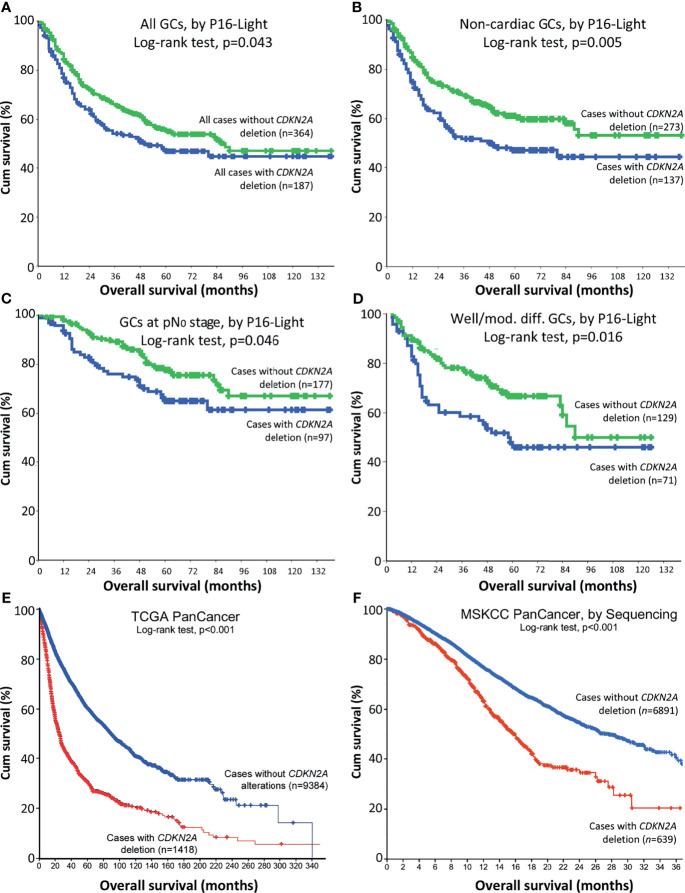
Relationship between *CDKN2A* deletion in cancer tissues and overall survival (OS) of patients in Kaplan-Meier analysis. **(A)** OS curves for merged patients with gastric carcinoma (GC) with and without *CDKN2A* deletion in P16-Light analysis. **(B)** OS curves for patients with non-cardiac GC with and without *CDKN2A* deletion. **(C)** OS curves for patients with non-lymph metastatic (pN_0_) GC with and without *CDKN2A* deletion. **(D)** OS curves for patients with well or moderately differentiated GC with and without *CDKN2A* deletion. **(E)** Overall survival curves for TCGA PanCancer patients with and without *CDKN2A* deletion, according to the datasets (29–31). **(F)** Overall survival curves for MSKCC PanCancer patients with and without *CDKN2A* deletion, according to the datasets (32, 41). Charts in **(E, F)** were adapted from images downloaded from the cBioport website.

To analyse the association between *CDKN2A* SCND and OS of GC patients, we emerged these data for the above 408 GC patients with those 156 patients enrolled in our WGS study together (25, 28). OS information was available for total 551 patients (**Data File 1**). In Kaplan-Meier analysis, OS of these GC patients (*n*=364) without *CDKN2A* SCND was significantly longer than those (*n*=187) with *CDKN2A* SCND (Cox univariate regression analysis: hazard ratio=0.767, 95% confidence interval=0.592-0.994; **Figure 2A**). Similarly, a significant association between *CDKN2A* SCND and OS was observed among GC patients in various sub-stratification groups (**Figures 2B–D**).

In addition, OS of pan-cancer patients (*n*=9384) without *CDKN2A* deletion was longer than those (*n*=1418) with *CDKN2A* deletion in TCGA project (*p*<0.001; **Figure 2E** and **Data File 2**) (6, 7, 31–33). OS of MSKCC PanCancer patients (*n*=6891) without *CDKN2A* deletion by target exon-captured deep sequencing was also longer than those (*n*=639) with *CDKN2A* deletion (*p*<0.001; **Figure 2F** and **Data File 3**) (34). These results suggest that *CDKN2A* SCND may be a poor survival factor not only for patients with GC, but also for patients with other kinds of cancers.

### *CDKN2A* SCND Promotes Migration and Invasion and Inhibits Apoptosis of Cells

**Figure 3:**
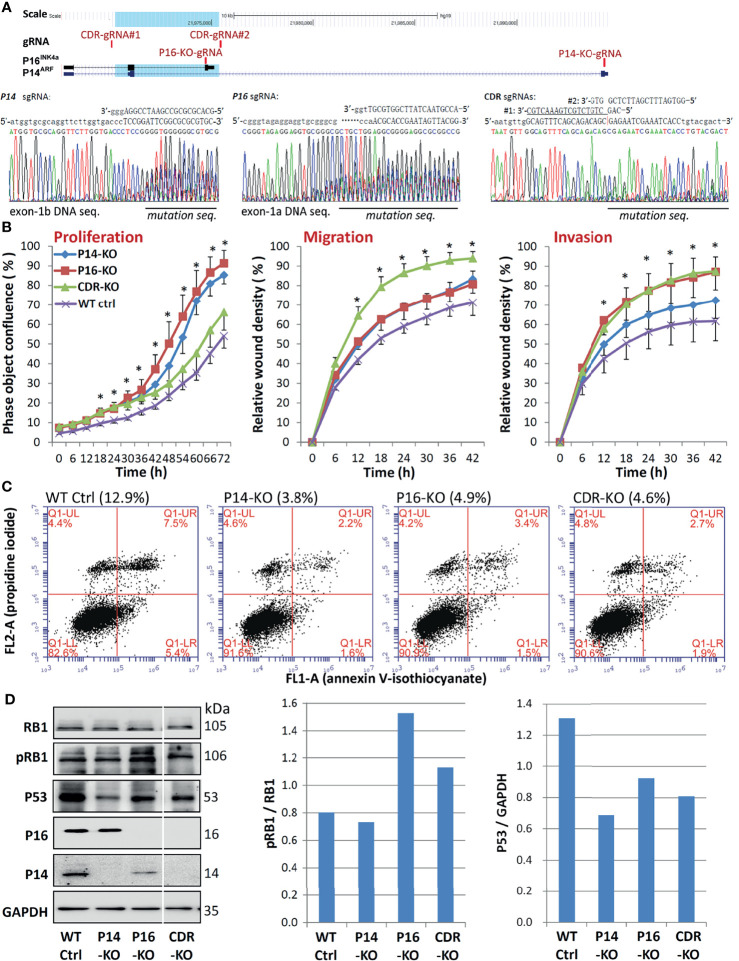
Comparison of behavioural analysis of MGC803 cells transfected with multiple *CDKN2A* KO genotypes. **(A)** CRISPR/Cas9 and corresponding single guide RNA (sgRNA) knockout (KO) of *CDKN2A* exon-1b, 1a, and CDR in cells. Locations of sgRNAs and exons are identified, and a blue shadow is used to show the 5.1-kb common deletion region (top chart). **(B)** Long-term dynamic IncuCyte studies were used to analyse the proliferation, migration, and invasion of pooled clones with various *CDKN2A* inactivation genotypes. Nine or twelve wells are used to calculate the average value of each point. It is also possible to view the standard deviation (SD). *P < 0.01 against *CDKN2A* wild-type control cells. **(C)** Using annexin V-isothiocyanate (FITC, FL1-A) and propidine iodide (PI, FL2-A) labeling, flow cytometry was utilized to evaluate the percentage of apoptotic versus dead cells in various *CDKN2A* inactivation genotypes. The percentages in parentheses represent the overall number of early and late apoptotic cells in various *CDKN2A* knockout genotypes. **(D)** The amounts of total RB1, phosphorylated RB1 (pRB1), P16, P14, proteins in cells with different *CDKN2A* KO genotypes was determined by Western blot analysis.

**Figure 4:**
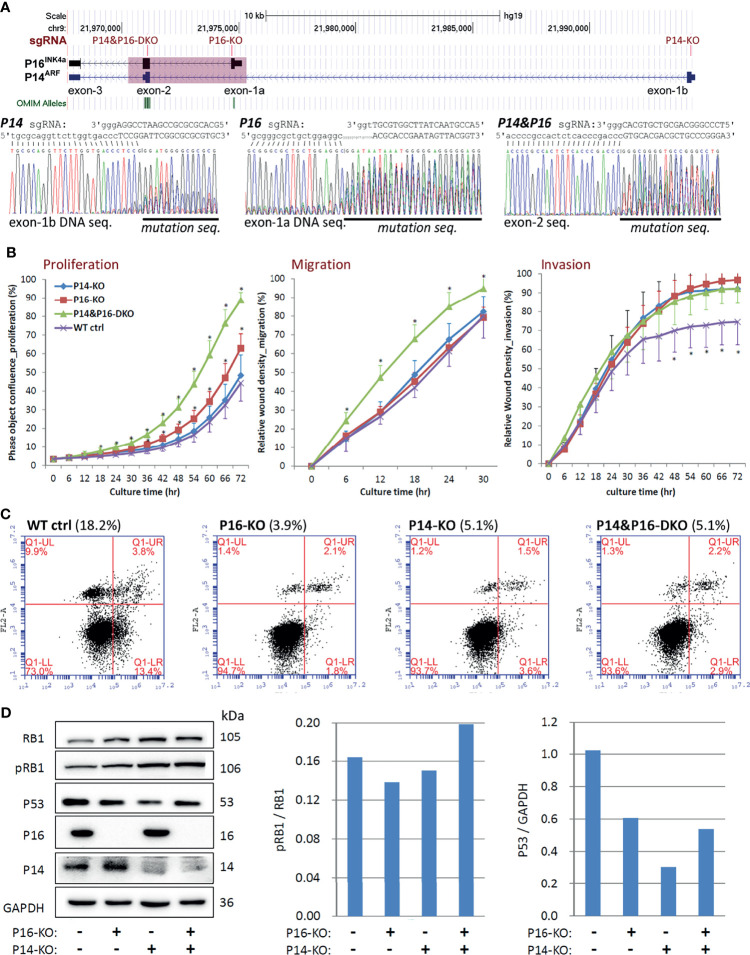
Comparison of behavioural effects of several *CDKN2A* knockout (KO) genotypes on HEK293T cells. **(A)** CRISPR/Cas9 and corresponding single guide RNA (sgRNA) knockout of *CDKN2A* exon-1b, 1a, and exon-2 in cells. The positions of exons and each sgRNA are identified, and a pink shadow is used to show the 5.1-kb common deletion region (top chart). **(B)** Long-term dynamic IncuCyte studies were used to analyse the proliferation, migration, and invasion of pooled clones with various *CDKN2A* inactivation genotypes. Each point represents the average value of nine or twelve wells. Additionally, the SD value is also displayed. *P < 0.01 against *CDKN2A* wild-type control cells. **(C)** Using annexin V-isothiocyanate (FITC, FL1-A) and propidine iodide (PI, FL2-A) labeling, flow cytometry was utilised to evaluate the percentages of apoptotic and dead cells in various *CDKN2A* inactivation genotypes. As indicated by the percentages in parentheses, the total number of early and late apoptotic cells in various *CDKN2A* knockout genotypes was calculated. **(D)** Western blot analysis was used to evaluate the quantities of total RB1, phosphorylated RB1 (pRB1), and P53 proteins in cells in various *CDKN2A* knockout genotypes.

The CDR overlaps with the *CDKN2A* exon-2 (27), which is a required exon for both *P16^INK4a^* and *P14^ARF^* (14). We further studied whether *P16^INK4a^* and *P14^ARF^* co-inactivation by *CDKN2A*-CDR deletion may play more roles in the development and progression of GCs than individual *P16^INK4a^* or *P14^ARF^* inactivation. Using CRISPR/Cas9, we were able to remove the exon-1a of *P16^INK4a^* (P16-KO), the exon-1b of *P14^ARF^* (P14-KO), and the common CDR of both *P16^INK4a^& P14^ARF^* (CDR-KO) in human MGC803 GC cells (**Figure 3A**). Two corresponding KO subclones were obtained and were pooled for each genotype and used to study their effects on alterations of cell behaviours. Long-term dynamic IncuCyte analysis showed that CDR-KO cells migrated and invaded the most among cells with different genotypes, as expected (**Figure 3B**). The proportion of apoptosis of MGC803 cells with various *CDKN2A* KO genotypes was only one third (34.3%) of that of *CDKN2A* wildtype (WT) cells (**Figure 3C**). The ratio of phosphorylated RB1 (pRB1) to total RB1 protein was higher in both CDR-KO and P16-KO cells than *CDKN2A* wildtype (WT) and P14-KO cells in Western blot analyses (**Figure 3D**). In contrast, the amount of P53 protein in these KO cells was much lower than that in the *CDKN2A* wildtype cells. Similar results were also observed in HEK293T cells with P14-KO, P16-KO, as well as *P16^INK4a^& P14^ARF^-*shared exon-2 (P14&P16-DKO) (**Figures 4A–D**). These results suggest that *CDKN2A* SCND may be a driver for GC development.

**Figure 5:**
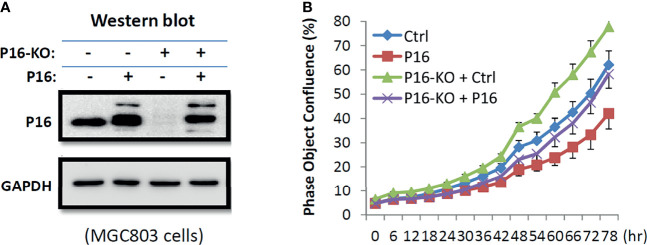
Effects of transient *P16^INK4A^* overexpression on the proliferation of MGC803 cells with and without P16-KO in the rescue experiment. **(A)** Image of Western blot; **(B)** The proliferation curves for cells with various states of *P16^INK4A^* function.

MGC803 cells were transiently transfected with a *P16^INK4A^* overexpression vector in order to determine if the increased cell proliferation is *P16^INK4A^* KO dependent. Long-term dynamic IncuCyte analysis showed that overexpression of *P16^INK4A^* greatly reversed the elevated proliferation phenotype of these cells, showing that the enhanced proliferation of P16-KO cells is *P16^INK4A^* inactivation-specific (**Figure 5**).

## Discussion

Hematogenous metastasis is the main recurrence route for GC patients after curative resection, which is different from lymphatic metastasis and direct peritoneal seeding. Hematogenous metastasis was associated with CD34-positive vessel density, vasculogenic mimicry, high VEGF-D or osteopontin expression (4, 5, 9, 12). Whether these factors drive hematogenous metastasis is not clear. Recently, it was reported that genetic inactivation of *Cdkn2a* by CRISPR/Cas9 promoted lung metastasis of mouse with non-small cell lung carcinoma transplanted subcutaneously (15). Through cross-sectional, prospective, and experimental studies, here, we reported that *CDKN2A* SCND was substantially correlated with hematogenous metastasis of GCs in both the cross-sectional and prospective studies. The results of our functional experiments further indicate that *CDKN2A* SCND could inhibit P53 expression and promote RB1 phosphorylation. *CDKN2A* inactivation also inhibited apoptosis and promoted proliferation/migration/invasion of cancer cells. These phenomena demonstrate that genetic *CDKN2A* inactivation may be a frequent causal factor and useful predictor for hematogenous metastasis of GCs.

Genetic inactivation of *CDKN2A* by SCND is very frequent in many human cancers (24), which is also associated with metastasis of cancers (23, 32–35). Our sub-stratification analyses using public TCGA datasets show that the relationship between *CDKN2A* deletion and cancer metastasis may be organ/tissue-specific. While *CDKN2A* deletion increases the risk of distant metastasis of HNSC, KIRC, PAAD, SKCM, and STAD/GC, it decreases the risk of distant metastasis of MESO. In addition, a strong relationship between *CDKN2A* deletion and distant metastasis was observed in cancers without lymphatic metastasis, but not in cancers with lymphatic metastasis. This is consistent with our current results observed in these patients enrolled in both the cross-sectional and prospective studies. The reasons accounted for these differences are worth further studying.

It is well known that tumor suppressor P53 is essential for cell apoptosis and oncogene MDM2 promotes degradation of P53 *via* protein ubiquitination (36). P53 mutations were reported as a driver of metastasis signalling pathways (37). Most circulating cancer cells die *via* PANoptosis, including anoikis, pyroptosis, apoptosis, and necroptosis, within the bloodstream (38, 39). Avoiding PANoptosis is essential for circulating cancer cells adhering to endothelial cells, extravasating and cloning in distant tissues. The activity of MDM2 is inhibited by P14^ARF^ protein within normal cells (40). As we reported recently (25), P14^ARF^ is co-inactivated in 92% of *CDKN2A*-deleted cancers. Both genetic and epigenetic inactivation of function of *CDKN2A* gene (*P16^INK4a^*, *P14^ARF^*, or both) inhibited apoptosis and senescence of human cells and promoted experimental lung metastasis of cancer cells (3, 15, 41). Once again, here, we found that knockout of *CDKN2A* CDR by CRISPR/Cas9 indeed decreased the amount of P53 protein and markedly inhibited the apoptosis of MGC803 GC and non-tumor HEK293T cells. The increased risk of hematogenous metastasis for patients with *CDKN2A* deleted GC is in line with these results.

In conclusion, we found that *CDKN2A* SCND was a frequent event in GC genomes and could be an useful predictor for hematogenous metastasis of GCs. *CDKN2A* SCND may be also a causal factor for distant metastasis of other cancers through decreasing cancer cell apoptosis and promoting the migration and invasion of cancer cells *via* downregulation of P53 expression and upregulation of RB1 phosphorylation. *CDKN2A* SCND leads to inactivation of both P16^INK4a^ and P14^ARF^ (two endogenous inhibitors for CDK4 and MDM2) in >90% *CDKN2A*-deleted cancers, it needs to study whether CDK4 and MDM2 inhibitor drugs could be used to prevent hematogenous metastasis of cancers.

## Data Availability Statement

The original contributions presented in the study are included in the article/**Supplementary Material**. Further inquiries can be directed to the corresponding authors.

## Ethics Statement

The Institution Review Board of Peking University Cancer Hospital & Institute approved this study and was carried out in accordance with the principles outlined in the Declaration of Helsinki. Informed consent was obtained from each patient prior to their inclusion in the study. The patients/participants provided their written informed consent to participate in this study.

## Author Contributions

All authors were involved in the study’s design and conception. JQ, YT, XC, ZL, JZ, LG, LZ, JJ, and RX prepared the materials, collected the data, and analysed it. DD, RX, and JQ wrote the original version of the paper, which was then revised by all authors.

## Funding

Funding for this project has been provided by grants to DD from the Beijing Natural Science Foundation (No. 7181002), the Capital’s Funds for Health Improvement and Research (No. 2018-1-1021), and the National Natural Science Foundation of China (No. 91640108).

## Conflict of Interest

The authors declare that the research was conducted in the absence of any commercial or financial relationships that could be construed as a potential conflict of interest.

## Publisher’s Note

All claims expressed in this article are solely those of the authors and do not necessarily represent those of their affiliated organizations, or those of the publisher, the editors and the reviewers. Any product that may be evaluated in this article, or claim that may be made by its manufacturer, is not guaranteed or endorsed by the publisher.

## Supplementary Material

Supplementary Figure 1 Relationship between *CDKN2A* deletion in TCGA PanCancer and the status of cancer local invasion, lymph metastasis, and distant metastasis in sub-stratification analyses. **(A)** The frequency of *CDKN2A* deletion in TCGA cancers without lymph metastasis; **(B)** The frequency of *CDKN2A* deletion in cancers of various histological subtypes without lymph metastasis; **(C)** The frequency of *CDKN2A* deletion in TCGA cancers with lymph metastasis; **(D)** The frequency of *CDKN2A* deletion in cancers of various histological subtypes with lymph metastasis. The exact numbers of cancer cases with and without *CDKN2A* deletion, total cancer cases, the frequency (%) of *CDKN2A* deletion in each subgroup of cancers are labeled, respectively. Significant *p*-values identified in a chi-square test are also listed. T_1-x_, local invasion stages; M_0_ and M_1-x_, distant metastasis-negative and distant metastasis -positive. BLCA, bladder carcinoma; BRCA, breast carcinoma; ESCA, esophagus carcinoma; HNSC, head and neck squamous cell carcinoma; LIHC, liver hepatocyte carcinoma; LUSC, lung squamous cell carcinoma; LUAD, lung adenocarcinoma; KIRC, kidney clear cell carcinoma; MESO, mesothelioma; PAAD, pancreas adenocarcinoma; SKCM, skin cutaneous melanoma; STAD, stomach adenocarcinoma.

## Abbreviations

CDR, common deletion region; GC, gastric carcinoma; OS, overall survival; CDR-KO, *P16^INK4a^& P14^ARF^-*shared CDR knockout; P14-KO: *P14^ARF^*-specific exon-1b knockout; P16-KO, *P16^INK4a^*-specific exon-1a knockout; P14&P16-DKO, *P16^INK4a^& P14^ARF^-*shared exon-2 knockout; SCND, somatic copy number deletion.
