# Supplementary material for: CDKN2A Deletion Leading to Hematogenous Metastasis of Human Gastric Carcinoma
Source: Front Oncol. 2021 Dec 23;11:801219. doi: 10.3389/fonc.2021.801219 (PMC8733704; doi:10.3389/fonc.2021.801219)
Supplement: Supplementary Figure 1 — Relationship between CDKN2A deletion in TCGA PanCancer and the status of cancer local invasion, lymph metastasis, and distant metastasis in sub-stratification analyses. (A) The frequency of CDKN2A deletion in TCGA cancers without lymph metastasis; (B) The frequency of CDKN2A deletion in cancers of various histological subtypes without lymph metastasis; (C) The frequency of CDKN2A deletion in TCGA cancers with lymph metastasis; (D) The frequency of CDKN2A deletion in cancers of various histological subtypes with lymph metastasis. The exact numbers of cancer cases with and without CDKN2A deletion, total cancer cases, the frequency (%) of CDKN2A deletion in each subgroup of cancers are labeled, respectively. Significant p-values identified in a chi-square test are also listed. T1-x, local invasion stages; M0 and M1-x, distant metastasis-negative and distant metastasis -positive. BLCA, bladder carcinoma; BRCA, breast carcinoma; ESCA, esophagus carcinoma; HNSC, head and neck squamous cell carcinoma; LIHC, liver hepatocyte carcinoma; LUSC, lung squamous cell carcinoma; LUAD, lung adenocarcinoma; KIRC, kidney clear cell carcinoma; MESO, mesothelioma; PAAD, pancreas adenocarcinoma; SKCM, skin cutaneous melanoma; STAD, stomach adenocarcinoma. [file Image_1.pdf]

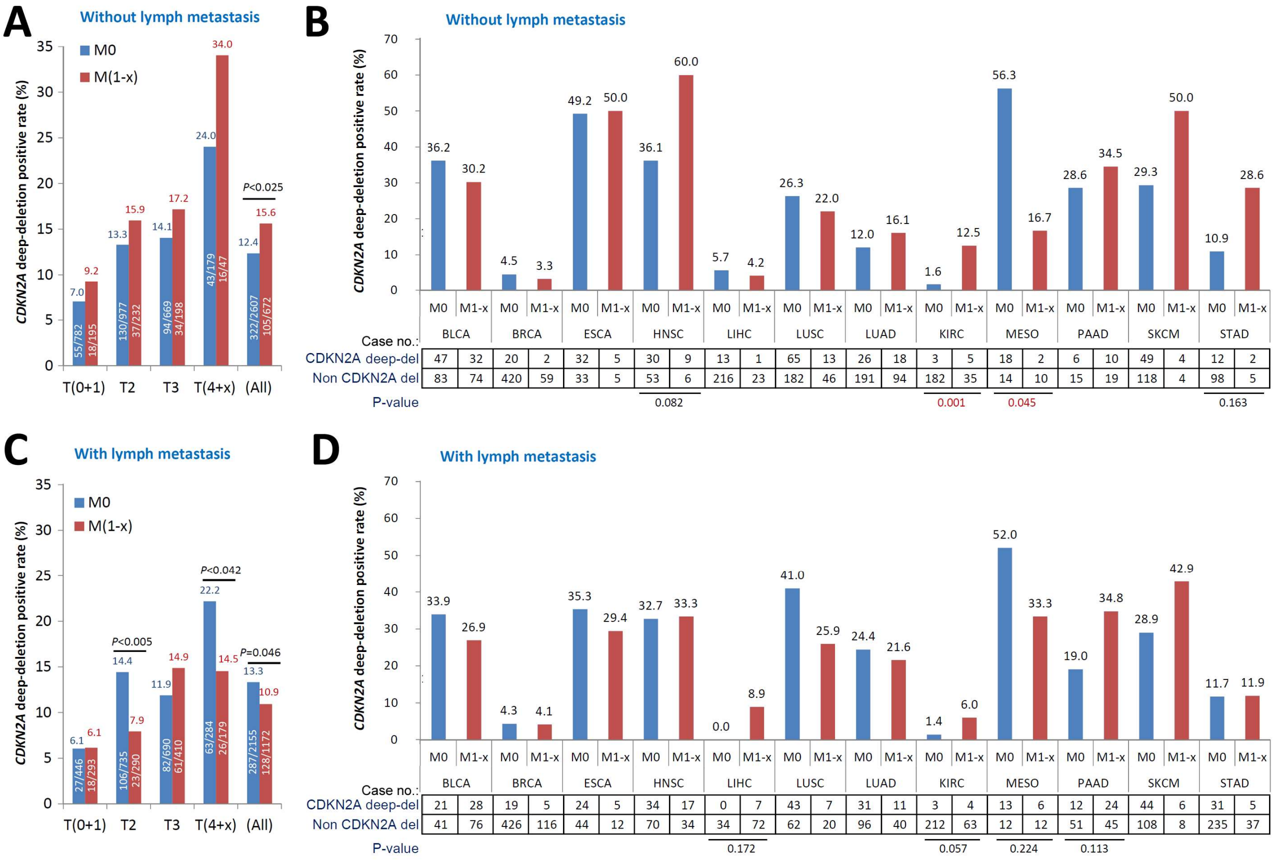

Figure S1. Relationship between *CDKN2A* deletion in TCGA PanCancer and the status of cancer local invasion, lymph metastasis, and distant metastasis in sub-stratification analyses. **(A)** The frequency of *CDKN2A* deletion in TCGA cancers without lymph metastasis; **(B)** The frequency of *CDKN2A* deletion in cancers of various histological subtypes without lymph metastasis; **(C)** The frequency of *CDKN2A* deletion in TCGA cancers with lymph metastasis; **(D)** The frequency of *CDKN2A* deletion in cancers of various histological subtypes with lymph metastasis. The exact numbers of cancer cases with and without *CDKN2A* deletion, total cancer cases, the frequency (%) of *CDKN2A* deletion in each subgroup of cancers are labeled, respectively. Significant *p*-values identified in a chi-square test are also listed. T_{1-x}, local invasion stages; M_0 and M_{1-x}, distant metastasis-negative and distant metastasis -positive. BLCA, bladder carcinoma; BRCA, breast carcinoma; ESCA, esophagus carcinoma; HNSC, head and neck squamous cell carcinoma; LIHC, liver hepatocyte carcinoma; LUSC, lung squamous cell carcinoma; LUAD, lung adenocarcinoma; KIRC, kidney clear cell carcinoma; MESO, mesothelioma; PAAD, pancreas adenocarcinoma; SKCM, skin cutaneous melanoma; STAD, stomach adenocarcinoma.
